# Contraction of the Lower Jaw

**Published:** 1846-09

**Authors:** A. Tamplin


*Contraction of the Lower Jaw.*—Sometime since I was consulted by a patient, aged 11 years, whose mouth was, and had been for two years rigidly closed. Upon introducing my finger in the angle of the mouth, a dense indurated cicatrix was found to exist on either side, which effectually prevented the patient from opening his mouth in the slightest degree. His mother stated that for the period mentioned he had lived by suction only. Many surgeons had been consulted, but all had given an unfavorable opinion. I determined to divide the cicatrices by a puncture externally, which was done in the following manner: The patient was laid on a table, the head being supported with a pillow, an assistant holding the head. I introtroduced a small scalpel perpendicularly upon the middle of the masseter muscle; then withdrew it, and inserted a long narrow blunt-pointed knife, carrying it down to the bone, depressing the handle so that I could pass it horizontally forwards, until I felt the point at the anterior edge of the cicatrix, to the outer side of the mucous membrane, which I was enabled to do by keeping the finger of my left hand within the angle of the mouth. I then turned the sharp edge towards it, and gradually divided the band transversely. By this proceeding the mucous membrane of the mouth was left uninjured, the cicatrix alone being divided; so that there was no possibility of hæmorrhage. Pledgets of lint were applied over the point of puncture, as well as over the line of incision, and secured with strapping and bandage. Scarcely any pain followed the operation; and, although only one cicatrix was divided, the boy could open his mouth to the extent of about the eighth of an inch.

Four days afterwards the puncture was healed, when I divided the cicatrix on the opposite side in a similar manner, and with a similar satisfactory result. At the end of four days following the last operation, I applied an instrument made to fit the teeth, of the upper jaw, which acted as a fulcrum, and with small narrow blunt steel hooks, which I introduced over the teeth of the lower jaw, and afterwards attached to the instrument by means of a screw, with which I gradually opened the mouth. During the night the instrument was removed, and small wedges of ivory were introduced, to which were attached pieces of tape, so that there should be no possibility of their getting into the mouth or throat. At the end of fourteen days, the boy was able to eat any kind of solid food, and, from his mother's account, was eating the whole of the day. He rapidly improved in health and strength, and, with a continuance of the use of the instrument, the cicatrices became gradually absorbed.—*Lectures on the Nature and Treatment of Deformities, by A. Tamplin.*

